# Interpreting psychological resilience in young African American women: a multilevel socioecological framework

**DOI:** 10.3389/fpubh.2026.1827741

**Published:** 2026-07-10

**Authors:** Sparkle Springfield-Trice

**Affiliations:** Department of Public Health Sciences, Parkinson School of Health Sciences and Public Health, Loyola University Chicago, Maywood, IL, United States

**Keywords:** African American women, cultural resources, public health, resilience, socioecological framework, stress, structural adversity, young adulthood

## Abstract

Resilience theory emphasizes that adaptive capacity must be understood in relation to adversity and the resources available for adaptation. However, public health research primarily interprets psychological resilience measures as indicators of individual adaptive capacity, with limited integration of sociological, historical, and developmental context. Such narrow interpretations of resilience may be especially consequential for young African American women, who experience disproportionately high exposure to stressors shaped by socioecological structures. This perspective proposes a socioecological framework to refine the interpretation of psychological resilience in public health research involving young African American women. Drawing on multisystem resilience theory, African-centered psychology, Indigenous health scholarship, community-engaged research, and prior empirical findings, this perspective proposes a socioecological framework that situates psychological resilience within patterned structural stress environments, culturally grounded resource systems, and life-course developmental contexts. Reframing the interpretation of resilience in public health research can strengthen methodological rigor and reduce oversimplified conclusions. Without this shift, narrow interpretations may promote detrimental narratives and lead to decontextualized interventions that are ineffective and unsustainable. Situating psychological resilience within multilevel systems of stress exposure and reciprocal resources supports culturally grounded research and informs the development of effective, multidimensional public health interventions.

## 1 Introduction

Resilience has become a central concept in the social sciences and medicine for understanding how individuals and populations navigate adversity. In psychology, resilience is defined as the capacity to access psychological, social, cultural, and physical resources that sustain well-being and to negotiate access to these resources in culturally meaningful ways (1). Sociological traditions similarly conceptualize resilience as survival and resistance within structurally constrained environments (2). Contemporary resilience theory, including multisystem and socioecological perspectives, views resilience as a dynamic process that emerges through interactions between stress exposure and adaptive resources across biological, psychological, social, and environmental systems (3, 4).

Despite these theoretical advances, most empirical research measures resilience as an individual psychological construct reflecting perceived adaptive capacity (5–9). These measures have demonstrated strong predictive value across multiple health domains, contributing to increased interest in resilience as a target for intervention. Although resilience is conceptualized in various ways across disciplines, psychological resilience measures remain the most widely used approach in epidemiological and public health research. Therefore, improving the interpretation of these measures is an important step toward advancing more contextually informed resilience research.

However, the empirical interpretation of psychological resilience often lags behind theoretical advances. Consequently, sociological dimensions of resilience—especially those conceptualized as adaptation, survival, and resistance within structurally constrained environments—are rarely considered when interpreting psychological resilience measures (10). This disconnect represents a significant limitation for public health research aiming to understand resilience within broader social and structural contexts (11, 12).

Narrow interpretations of resilience may be particularly consequential for African American (AA) women, whose lives are shaped by both culturally grounded resources and structurally patterned stressors (13–17). Black feminist scholars have long emphasized that AA women navigate intersecting systems of oppression in which racism and sexism operate simultaneously rather than independently (18, 19). Gendered racism is a structural force that shapes both access to resources and the psychological processes central to resilience. Embedded within social systems, it influences how individuals anticipate stress, appraise environmental threats, and mobilize adaptive resources (20). These resources may include identity affirmation and positive self-concept, as well as community-based supports such as mentorship networks, economic opportunities, and other culturally grounded structures that facilitate adaptation (21, 22).

AA women have sustained collective survival through dynamic engagement with both inherited and emergent resource systems. Longstanding traditions of cultural resilience—including spirituality, self-definition, and collective responsibility—have been maintained and adapted across generations, enabling navigation of environments shaped by intersecting systems of oppression (19). In this context, resilience reflects not only individual coping but also the ongoing reconfiguration of culturally grounded and context-specific resources in response to shifting demands.

Comprehensive interpretation of resilience is particularly salient among African American women in young adulthood, a developmental period marked by major social, institutional, and economic transitions. As young African American women move through higher education, workforce entry, family planning, and evolving social roles, they must interpret stressors while simultaneously renegotiating access to and mobilizing both traditional and newly available resources (23). Moreover, as these transitions unfold within the enduring influence of multigenerational gendered racism, residual constraints—including internalized stigma, limited economic opportunity, constrained reproductive decision-making, and caregiving responsibilities—may shape how stress is interpreted and how resources are discerned (24–27). These dynamics may blur the boundaries between stressors and adaptive resources, reflecting Du Bois’ conceptualization of double consciousness (28). As a result, resilience during young adulthood is not merely a process but a contextually contingent system of meaning-making and resource identification, access, and mobilization within structurally patterned environments.

Advancing resilience research in YAAW requires attention to two foundational principles of resilience theory (3–5). First, resilience must be evaluated in relation to adversity and positionality. The multidimensional stress environments experienced by YAAW should be specified and contextualized when interpreting psychological resilience scores in this group. Second, resilience is a multilevel process shaped by access to psychological, social, and cultural resources. Extending multisystem resilience theory, this perspective emphasizes that resilience is also shaped by how these resources are historically situated, interpreted, and recognized within structurally patterned environments. Yet, research rarely examines how resilience assessments capture these interpretive processes within the lived experiences of YAAW.

## 2 Objectives

**Figure 1:**
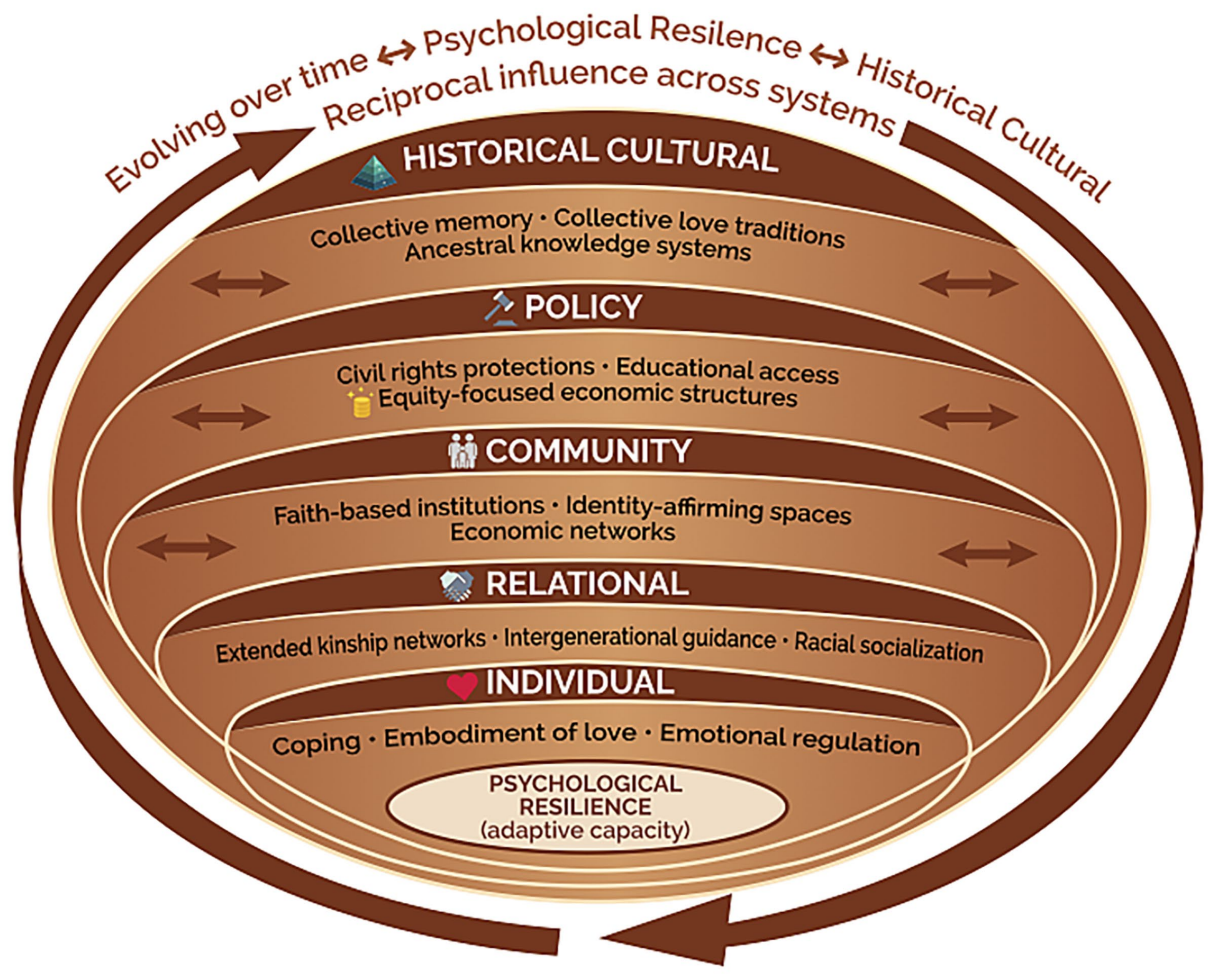
Multilevel reciprocity framework of resilience. This figure presents a socioecological model of resilience processes in young African American women. Psychological resilience is shaped by reciprocal interactions among individual, relational, community, policy, and historical–cultural contexts. In this framework, psychological resilience scores serve as observable indicators of adaptive capacity, with their interpretation contingent on the broader structural, cultural, and developmental environments in which they are generated. The individual layer reflects biological regulation, psychological coping, and the embodiment of identity affirmation or spirituality. The relational layer highlights interpersonal support systems, including family networks, mentorship, and peer relationships. The community layer represents collective environments that promote belonging and resource sharing. The policy layer reflects structural conditions that shape opportunities and provide protection from discrimination. The historical–cultural layer captures collective memory, cultural continuity, and ancestral traditions that influence resilience across generations. Bidirectional arrows indicate reciprocal influence across levels, reflecting the dynamic exchange of resources, knowledge, care, and support among individual, relational, community, policy, and historical–cultural systems. These reciprocal processes include the transmission of cultural knowledge, collective responsibility, intergenerational support, and traditions of collective love and care that shape resilience across generations. The outer circular arrow represents ongoing feedback processes through which individuals and communities identify, access, mobilize, and sustain culturally grounded resources over time.

To address the interpretive gaps described above, this perspective introduces the Multilevel Reciprocity Framework of Resilience, a socioecological approach for interpreting psychological resilience in YAAW. Drawing on critiques of resilience scholarship, empirical evidence, African-centered psychology, and Indigenous health research, the framework identifies key contextual domains that can shape how resilience develops, is expressed, and is evaluated. As illustrated in Figure 1, psychological resilience is situated within interacting individual, relational, community, policy, and historical–cultural environments.

Rather than proposing a new theory, the framework identifies domains that researchers may consider when interpreting psychological resilience scores and emphasizes reciprocal influences across systems and generations. This approach may help address the limitations of overly narrow interpretations. Examining these critiques is therefore an important step in understanding why a more contextually grounded framework for interpreting resilience is needed.

## 3 Critique of resilience research

A substantial body of scholarship has examined resilience in AA women, highlighting important limitations in how resilience is conceptualized and interpreted. Woods-Giscombé and colleagues document considerable definitional variability across disciplines and emphasize that resilience research frequently centers on individual coping, with limited integration of structural and cultural context (29). Although this work clarifies how resilience has been defined across fields, it does not fully resolve how psychological resilience measures should be interpreted when individuals are navigating patterned structural stress exposure.

Additional scholarship cautions that resilience narratives may inadvertently reinforce expectations of emotional endurance AA women and girls. Bentley-Edwards and Adams (30) argue that cultural archetypes such as the “Superwoman,” “Black Girl Magic,” or “beautiful struggle” may normalize emotional suppression and obscure the structural conditions that produce persistent stress exposure. The superwoman schema literature further demonstrates that expectations of strength and self-sacrifice can carry physiological costs (31). Similarly, Brody and colleagues’ work on “skin-deep resilience” shows that strong psychosocial functioning can coexist with physiological strain under chronic stress conditions (32). Living under prolonged emotional strain may contribute to adverse health outcomes, consistent with research on high-effort coping and cumulative physiological burden (33, 34).

Although psychological resilience scores can serve as valuable indicators, evidence such as the “skin-deep resilience” phenomenon—where high psychological resilience coexists with physiological strain—demonstrates that these scores cannot fully capture the complexity of resilience processes without accounting for broader contextual and patterned stress environments.

Overall, these findings demonstrate that oversimplified interpretations of resilience can obscure the interplay among structural stress exposure, cultural expectations of endurance, physiological burden, and reciprocal individual–structural processes. Such simplification may reinforce harmful expectations of endurance and mask underlying strain, highlighting the need for more contextually grounded interpretations.

## 4 Empirical evidence informing resilience interpretation

This section reviews key empirical studies that illustrate how resilience processes operate in African American women and how these findings inform the interpretation of psychological resilience measures. Empirical research demonstrates that resilience processes are observable but context dependent. Medical epidemiologic scholarship has documented resilience processes within AA populations and emphasized the importance of examining adaptive mechanisms alongside cardiovascular disease risk factors (35). Developmental research similarly shows that culturally grounded resources, such as racial socialization and community-based support systems, shape resilience processes in AA youth and young adults (36).

Analyses from the Women’s Health Initiative (mean age approximately 77 years) suggest that these dynamics persist across the life course. Springfield et al. (37) found that resilience, as measured by the Brief Resilience Scale, was associated with access to both psychosocial and non-psychological resources, and that race and ethnicity modified these relationships. AA women reported a higher average resilience than other racial and ethnic groups. However, higher resilience was not associated with greater stress exposure; instead, it was linked to fewer reported stressors and greater access to resources.

These findings highlight the importance of resource access in sustaining resilience later in life. A separate study found that higher resilience was associated with health-promoting behaviors, including greater odds of achieving recommended sleep duration. However, race-based differences in sleep persisted, and this association was not observed in older AA women, suggesting that the health benefits of resilience are not uniformly realized across subgroups (38). Our most recent research in YAAW (mean age approximately 24 years) demonstrates that culturally grounded resources are positively associated with psychological resilience, whereas culturally informed stressors, including internalized racism and adverse childhood experiences, are negatively associated with resilience (39).

Together, these findings suggest that psychological resilience scores reflect processes operating across individual, relational, and community dimensions, and that they are interpreted in relation to the dynamic stress environments and resource systems in which they are produced (Figure 1).

## 5 African-centered psychology and multidimensional frameworks

To further contextualize the empirical findings discussed above, this section examines theoretical perspectives that inform a multidimensional understanding of resilience in young African American women. African-centered psychology provides a theoretical foundation for understanding the multidimensional influences shaping mental health and resilience in people of African descent by situating psychological analysis within African cultural values, historical experiences, and collective systems of identity and wellbeing (40–43). Accordingly, it offers a useful interpretive lens for understanding what psychological resilience scores may represent within culturally and historically structured contexts. In African-centered psychology, psychological health is understood to arise from cultural systems of meaning that emphasize self-knowledge, cultural continuity, and communal responsibility. This perspective highlights the importance of collective resource systems—including community networks, shared identity, and cultural knowledge—in shaping adaptive functioning and wellbeing (42).

Within this framework, cultural identity, historical awareness, and community traditions shape psychological access to resources. These factors affect how individuals recognize and utilize available sources of support, which may, in turn, contribute to psychological resilience. From this perspective, resilience scores may reflect culturally grounded resources embedded in community relationships, cultural practices, and shared histories (40–43).

Similar insights have been articulated within Indigenous health and resilience scholarship, which emphasizes cultural continuity, collective identity, relational systems of well-being, and the restoration of traditional knowledge systems, while foregrounding the role of colonial trauma in shaping resilience processes (44, 45). These studies provide a critical lens for understanding the positionality of YAAW and for interpreting psychological resilience measures within this group, particularly regarding how they interact with perceived stress, access culturally grounded resources, and achieve health outcomes. For example, awareness of historical and ongoing gendered anti-Black racism shapes how YAAW interpret experiences of stress, situating these experiences within broader structural contexts rather than viewing them as individual deficits. Similarly, culturally grounded resources—such as community support networks, intergenerational knowledge, and shared cultural identity—influence how YAAW recognize and mobilize adaptive responses to stress, which may, in turn, be reflected in resilience scores.

Building on this, Indigenous frameworks broadly emphasize the role of history in shaping well-being and how knowledge and interpretation of that history inform lived experience and identity. These perspectives offer important conceptual tools for interpreting the lived experiences and historical positionality of YAAW. They highlight resilience as a dynamic, embodied process shaped by interactions between individual and structural systems over time and linked to collective memory and historical context (44, 45), as reflected in Figure 1.

## 6 Lessons from indigenous scholarship

Chandler and Lalonde demonstrated that cultural continuity is associated with lower suicide rates in First Nations communities (46). Subsequent Indigenous health scholarship has conceptualized resilience as emerging from relationships rooted in cultural identity, community self-determination, and intergenerational knowledge transmission, rather than solely from individual coping capacities (44, 47).

Indigenous scholars have emphasized the importance of interpreting health and resilience within the context of colonial histories and ongoing structural conditions. Frameworks such as the Indigenist Stress-Coping Model situate well-being within interacting systems of historical trauma, cultural identity, and culturally grounded coping resources (45). Yellow Horse Brave Heart highlighted the intergenerational psychological consequences of colonial trauma, and more recent community-engaged research has extended these insights to young adults (48), highlighting the importance of sovereignty, identity, and participatory methods in cultivating resilience (49). In parallel, emerging Black–Indigenous scholarship has highlighted how African and Indigenous histories intersect through shared experiences of colonial dispossession, cultural survival, and resistance (50–52). Central to recognizing the existing resilience of YAAW, Black Indigeneity frameworks emphasize the disrupted yet reconstituted relationships to land, memory, and community that characterize the historical experiences of AAs.

These perspectives further clarify how psychological resilience measures may reflect an individual’s awareness of, and psychological and tangible access to, relational and historical–cultural resources, including identity, community networks, and shared systems of meaning. Most importantly, they highlight the reciprocal and dynamic nature of these relationships, illustrating the connection between historical awareness and individual psychological processes, as well as how interactions across individual, relational, and community levels both shape and are reshaped by the lived experiences of YAAW over time (50, 51) (see arrow patterns in Figure 1).

African-centered and Indigenous scholarship emphasize the importance of interpreting resilience processes within culturally grounded systems of meaning and historical context. When considered alongside empirical findings on psychological resilience, these perspectives may clarify how YAAW recognize, interpret, and mobilize resources in response to ongoing multidimensional stressors. However, empirical public health research offers limited guidance for interpreting psychological resilience scores within dynamic, multidimensional environments. Together, these bodies of work support a structured, multilevel approach to interpreting psychological resilience (Figure 1).

## 7 Application of the multilevel reciprocity framework

Building on these insights, the Multilevel Reciprocity Framework of Resilience, an asset-based socioecological approach, situates psychological resilience in YAAW within structural stress environments, culturally grounded resource systems, and life-course contexts. It conceptualizes resilience as a multilevel adaptive process shaped by reciprocal interactions across individual, relational, community, policy, and historical–cultural domains. By centering culturally grounded assets, the framework provides a strengths-based perspective for interpreting resilience literature and advancing more contextually grounded public health research in YAAW.

### 7.1 Individual-level processes

At the individual level, resilience scores may reflect biological, psychological, and spiritual processes that influence how individuals interpret stress exposure and regulate emotional responses. These processes may include the biological embodiment of stress (32, 53–56), coping strategies (57–59), and culturally grounded strengths such as identity affirmation, spirituality, belonging, and traditions of collective love and care emphasized in African-centered psychology, which signal cultural continuities and ancestral systems of meaning (42, 60–62). These processes reflect the reciprocal relationship between individual functioning and broader historical and cultural systems, as illustrated in Figure 1.

### 7.2 Relational-level processes

The relational layer highlights interpersonal environments that may shape resilience processes. These include extended kinship networks, which reinforce collective responsibility and social support (63, 64); mentorship and teacher guidance, which support identity development and educational navigation (65); and peer and social support, which can buffer stress and promote adaptive coping in AA women (66, 67). Intergenerational relationships, particularly those involving mothers, grandmothers, and other elders, often transfer cultural knowledge, coping strategies, and guidance for navigating adversity across generations (19).

Relational contexts may also reflect broader intersecting experiences of racism and sexism that shape family dynamics, interpersonal trust, and expectations regarding identity, emotional expression, and caregiving (15, 19, 68). For example, AA families may engage in racial and gendered socialization practices that prepare young women to navigate bias, discrimination, and stereotypes while reinforcing cultural pride, emotional strength, self-definition, and collective responsibility. These messages may influence how trust is developed and extended across institutional and social environments (68). Internalized messages about race, gender, and social positioning may also shape how individuals view themselves, interpret others’ intentions, and negotiate belonging (15, 69). In some cases, internalized racism and gendered stereotypes may contribute to social distancing or mistrust in interpersonal relationships. In other cases, shared experiences of marginalization may promote solidarity, mutual affirmation, and the development of deep relational bonds (e.g., sisterhood) (19). Collectively, these relational processes may influence how AA women anticipate bias, interpret social interactions, access support, and navigate belonging.

### 7.3 Community-level processes

The community layer reflects collective environments that may shape resilience processes through shared identity, belonging, leadership development, and resource exchange. These environments include community institutions and organizations such as churches, sororities, women’s organizations, and professional networks, including the Association of Black Psychologists (70–72). Participation in community-based and identity-affirming spaces has been linked to increased social support, belonging, and mental health among AA adolescents and college students (73–75). Historically, these institutions have served as sites of cultural affirmation, mentorship, leadership development, and collective support for AA women and girls.

For example, guidance from mothers, grandmothers, and other community elders often provides YAAW with practical strategies for navigating schools, workplaces, healthcare settings, and other institutions (19, 63). Studies of racial socialization show that messages emphasizing cultural pride and preparation for bias help Black youth develop coping strategies for racialized stressors and are associated with improved psychological adjustment and other resilience-related factors (76, 77). Such guidance may be transmitted through everyday conversations, church-based youth programs, mentoring relationships, and other community spaces, helping YAAW interpret challenges and identify available resources. In addition to providing practical guidance, these intergenerational exchanges may also transfer historical and cultural knowledge that helps YAAW understand contemporary challenges, including intersecting forms of racism and sexism (sometimes described as misogynoir), within broader social and historical contexts (78).

AA women have also played central roles in sustaining community-based adaptive systems of care, resistance, and resource sharing across both historical and contemporary contexts. These systems include church-based mutual aid, women’s club organizations, informal “othermothering” and shared childcare, workplace and neighborhood networks, and everyday social spaces such as beauty salons, laundromats, and home kitchens (19, 79–82). These longstanding forms of community engagement continue to evolve, with digital technologies and social media providing new avenues for connection, community-building, and resource exchange. For example, online platforms have expanded opportunities for Black-owned businesses and entrepreneurial ecosystems to reach broader audiences and have enabled the emergence of identity-affirming spaces that support cultural expression, community connection, and psychological well-being (19, 22, 83).

### 7.4 Policy-level influences

The policy layer reflects structural conditions that shape exposure to stress, access to resources, and opportunities for adaptation across the life course. These conditions include laws, policies, and institutional practices that influence access to economic opportunity, education, healthcare, housing, and political participation. One understudied example is the large-scale loss of AA-owned farmland during the twentieth century. African American farmers owned more than 16 million acres of land by 1910, but discriminatory lending practices and heirs’ property vulnerabilities contributed to dramatic land loss, the effects of which remain visible today (84, 85). Given that land ownership remains an important source of intergenerational wealth, these policy-driven losses illustrate how structural conditions shape the transmission of material resources across families and communities.

More broadly, historical and contemporary systems—including enslavement, segregation, discriminatory labor conditions, housing inequities, educational exclusion, and unequal access to quality healthcare—have shaped patterns of resource distribution and opportunity across generations (82, 86, 87). Notably, these systems often operated through intersecting forms of racial and gender stratification that influenced AA women’s access to economic resources, political participation, educational opportunities, and institutional power. Nonetheless, AA women have also contributed to shaping policy environments through civil rights organizing, educational advocacy, and economic justice efforts.

For example, Septima Poinsette Clark’s citizenship schools expanded civic participation and supported voting rights efforts, Ella Baker’s organizing strategies strengthened grassroots movements that influenced civil rights policy implementation, and Fannie Lou Hamer’s advocacy challenged political exclusion and brought national attention to voting rights enforcement (88–90).

Importantly, these effects extend beyond economic resources. The loss of land, community spaces, and lives has contributed to forms of cultural bereavement that may shape perceptions of opportunity, security, belonging, and resource access among YAAW today (91, 92). Recognizing these policy contexts and their intergenerational consequences also highlights how AA women have contributed to expanding access to rights, protections, and resources across generations.

### 7.5 Historical and cultural contexts

The historical–cultural layer reflects systems of meaning, memory, and knowledge that shape resilience across generations. These systems include survival traditions, cultural continuity and identity, and ancestral knowledge grounded in African intellectual traditions and community wisdom (42, 93, 94). African-centered youth development programs demonstrate how culturally grounded socialization can support identity development and well-being, while African intellectual traditions emphasizing wisdom, resistance, and self-knowledge illustrate how adaptive strategies are transmitted across generations (95, 96).

For AA women, cultural transmission has often occurred through everyday practices that preserve identity, strengthen community, and sustain collective survival. Traditions such as foodways, gardening, and intergenerational gatherings serve as practices of cultural transmission that reinforce collective memory and transmit adaptive knowledge across generations (63, 64, 97–99). Positive racial identity and continuity have been associated with improved psychological well-being and resilience among AA youth and women, with racial identity functioning as a protective factor against negative mental health outcomes (100, 101). These practices create opportunities for elders to share lessons learned and prepare younger generations to navigate adversity.

Walker’s discussion of “our mothers’ gardens” extends this perspective by illustrating how creativity, spirituality, caregiving, storytelling, and artistic expression may serve as culturally transmitted resilience resources among African American women despite longstanding structural oppression (102). Historically rooted practices that persist in contemporary forms of care and well-being include the preservation of herbal remedies and African-derived medical knowledge, midwifery, and everyday self-care practices such as skin and hair care. Through these forms of expression, AA women have maintained cultural memory, collective care, and systems of meaning across generations, which may be reflected in the psychological resilience of YAAW today.

These historical and cultural influences continue to shape and evolve, affecting contemporary forms of meaning, connection, and resilience. As noted previously, digital spaces have become important arenas where AA women build community, promote counter-narratives to gendered anti-Black racism, and expand opportunities for connection and resource exchange. Movements such as The Nap Ministry frame rest and healing as forms of resistance to chronic racial stress (103, 104). Emerging Black and Indigenous scholarship further highlights shared histories of cultural survival, resistance, and collective memory (50, 51). Together, these systems of meaning and cultural transmission may influence how YAAW interpret stress, identity, and support, suggesting that resilience reflects engagement with culturally grounded systems of knowledge, memory, and survival.

## 8 Implications of the multilevel reciprocity framework

The following subsections note implications for individuals, families, research, and policy.

### 8.1 Implications for individuals and families

YAAW and their families may benefit from understanding resilience as a process shaped by both individual coping strategies and broader systems of support. Awareness of resilience processes may help individuals recognize how practices such as stress regulation, identity affirmation, mentorship, spirituality, and engagement in culturally affirming spaces contribute to well-being. Families, mentors, and community institutions can reinforce these resources by creating environments that promote belonging, cultural identity, collective responsibility, and problem-solving.

### 8.2 Implications for research

This framework may serve as an interpretive guide for researchers engaging with both existing and future resilience literature. It does not suggest that psychological resilience measures should be abandoned or replaced; rather, it encourages researchers to interpret resilience scores within the broader social, cultural, developmental, and structural environments in which they are produced. This approach may help address longstanding critiques that resilience research often overemphasizes individual adaptation while overlooking the resource systems, historical contexts, and patterned stress environments that shape resilience processes.

Although developed with YAAW in mind, the framework may also offer a useful lens for interpreting resilience in older AA women and other populations whose lived experiences are shaped by identity-related stressors, historical marginalization, or disruptions to cultural, social, and community resources. By encouraging researchers to consider both adversity and access to resources, the framework may support more contextually grounded interpretations of resilience findings across diverse populations.

Public health researchers may be encouraged to engage in multidisciplinary team science and incorporate unique social predictors in future epidemiological studies. Integrating psychological resilience measures with assessments of gendered racism, social support networks, cultural resources, and community conditions can provide a more comprehensive understanding of the environments in which resilience develops. Longitudinal designs may be especially valuable for examining how resilience processes change across developmental transitions, such as entry into college, workforce participation, parenthood, and aging.

Mixed-methods approaches may further strengthen resilience research by combining quantitative measures with qualitative exploration of lived experience (105). For example, resilience scale scores can be examined alongside qualitative accounts in a joint display to explore how participants interpret stress and draw on cultural and relational resources in their daily lives. Qualitative interviews and focus groups can illuminate how participants define resilience, resistance, flourishing, caregiving, emotional strength, identity, and collective support from their own perspectives. These approaches may help researchers better understand how individuals interpret resilience processes, navigate stress across environments, and assign meaning to culturally grounded coping practices that may not be fully captured through resilience measures alone.

Research on culturally grounded community programs for AA youth suggests that psychosocial development is strengthened when interventions are community-engaged, culturally relevant, and reflective of AA lived experiences and traditions (106). Mixed-methods and participatory approaches may be particularly valuable for studying complex health and developmental processes because they integrate quantitative indicators with open-ended accounts of lived experience, generating insights that may not emerge from either approach alone (107). These approaches can reveal resilience resources, forms of resistance, and pathways to flourishing that are not readily visible through resilience scores alone, thereby generating new hypotheses, intervention targets, and culturally grounded theories of adaptation.

Community-engaged research approaches, including partnerships, advisory boards, and participatory frameworks, can help ensure that resilience constructs and measures reflect lived experiences and culturally grounded understandings of coping and well-being. High-quality community-engaged partnerships often require substantial time, trust building, reciprocal collaboration, shared decision-making, equitable compensation, and sustained engagement with community leaders and participants throughout the research process. Such investments may be especially important in research involving AA women, given longstanding histories of structural inequity, medical mistrust, and deficit-oriented interpretations of AA health research and development. Robust community-engaged partnerships may therefore strengthen contextual understanding, improve interpretive validity, and support more equitable and culturally grounded resilience research.

### 8.3 Implications for policy

The ideas discussed in this perspective also suggest practical policy applications for strengthening environments that support resilience. Educational institutions can implement mentorship programs that connect YAAW with faculty, professionals, and community leaders who offer guidance and social support. Policies that support community-based organizations, culturally affirming spaces, and youth development initiatives may enhance the social and cultural resources that facilitate adaptation.

Housing, economic development, and educational policies that increase access to stable housing, homeownership, entrepreneurship, quality education, and community resources may also strengthen the broader environments in which resilience develops. From this perspective, resilience-promoting policy extends beyond individual behavior change to include investments in the social, economic, and cultural conditions that support long-term well-being.

## 9 Conclusion

This perspective refines the interpretation of psychological resilience in public health research involving young African American women (YAAW) by situating resilience within cultural, historical, developmental, and structural contexts. YAAW navigate environments shaped by persistent stress exposure as well as culturally grounded resource systems, and interpreting resilience without considering these contexts may obscure the relational networks, community resources, cultural traditions, and adaptive strategies that support their well-being.

To support more contextually grounded interpretation, this paper proposes the Multilevel Reciprocity Framework of Resilience, an asset-based socioecological model that situates resilience within reciprocal interactions across individual, relational, community, policy, and historical–cultural systems. Within this framework, psychological resilience scores serve as indicators whose meaning depends on the broader environments in which they are produced and provide a starting point for examining resilience processes and identifying potential points of intervention. Importantly, this perspective extends beyond how resilience is interpreted in research contexts to how YAAW themselves understand and engage their resilience. These interpretive processes may support health-promoting practices, including the establishment of healthy boundaries, recognition of adaptive strengths, and the cultivation of meaning, memory, and reconnection.

Focusing on resilience as a multilevel and contextually contingent system may help clarify how YAAW identify, access, and mobilize resources across contexts, contributing to a more nuanced interpretation of existing research. Advancing this work may benefit from approaches that center context, such as interdisciplinary collaboration, community-engaged research, and mixed-methods designs. Although this framework is grounded in existing literature and reflects the limited empirical evidence base on resilience among YAAW, it provides a foundation for hypothesis generation and underscores the need for future research that empirically examines resilience as a multilevel, contextually situated, and interpretive process. Such work is potentially important to refining measurement, testing theoretical assumptions, and informing interventions that effectively support the health and wellbeing of YAAW. Together, these efforts provide a foundation for continued study of resilience among YAAW in ways that are contextually grounded and equity focused.

## Data Availability

The original contributions presented in the study are included in the article/supplementary material, further inquiries can be directed to the corresponding author.
